# Deep Learning Using Preoperative AS-OCT Predicts Graft Detachment in DMEK

**DOI:** 10.1167/tvst.12.5.14

**Published:** 2023-05-15

**Authors:** Alastair Patefield, Yanda Meng, Matteo Airaldi, Giulia Coco, Sabrina Vaccaro, Mohit Parekh, Francesco Semeraro, Kunal A. Gadhvi, Stephen B. Kaye, Yalin Zheng, Vito Romano

**Affiliations:** 1 Department of Eye and Vision Sciences, Institute of Life Course and Medical Sciences, University of Liverpool, Liverpool, UK; 2 Department of Molecular and Translational Medicine, University of Brescia, Brescia, Italy; 3 Department of Corneal Diseases, St. Paul's Eye Unit, Liverpool University Hospitals NHS Foundation Trust, Liverpool, UK; 4 Department of Clinical Science and Translational Medicine, University of Rome Tor Vergata, Rome, Italy; 5 Department of Ophthalmology, University of “Magna Graecia,” Catanzaro, Italy; 6 Schepens Eye Research Institute, Massachusetts Eye and Ear, Department of Ophthalmology, Harvard Medical School, Boston, MA, USA; 7 Ophthalmology Clinic, Department of Medical and Surgical Specialties, Radiological Sciences, and Public Health, University of Brescia, Brescia, Italy; 8 Liverpool Centre for Cardiovascular Science, University of Liverpool and Liverpool Heart and Chest Hospital, Liverpool, UK

**Keywords:** DMEK, artificial intelligence, predictive factor, rebubbling

## Abstract

**Purpose:**

To evaluate a novel deep learning algorithm to distinguish between eyes that may or may not have a graft detachment based on pre–Descemet membrane endothelial keratoplasty (DMEK) anterior segment optical coherence tomography (AS-OCT) images.

**Methods:**

Retrospective cohort study. A multiple-instance learning artificial intelligence (MIL-AI) model using a ResNet-101 backbone was designed. AS-OCT images were split into training and testing sets. The MIL-AI model was trained and validated on the training set. Model performance and heatmaps were calculated from the testing set. Classification performance metrics included F1 score (harmonic mean of recall and precision), specificity, sensitivity, and area under curve (AUC). Finally, MIL-AI performance was compared to manual classification by an experienced ophthalmologist.

**Results:**

In total, 9466 images of 74 eyes (128 images per eye) were included in the study. Images from 50 eyes were used to train and validate the MIL-AI system, while the remaining 24 eyes were used as the test set to determine its performance and generate heatmaps for visualization. The performance metrics on the test set (95% confidence interval) were as follows: F1 score, 0.77 (0.57–0.91); precision, 0.67 (0.44–0.88); specificity, 0.45 (0.15–0.75); sensitivity, 0.92 (0.73–1.00); and AUC, 0.63 (0.52–0.86). MIL-AI performance was more sensitive (92% vs. 31%) but less specific (45% vs. 64%) than the ophthalmologist's performance.

**Conclusions:**

The MIL-AI predicts with high sensitivity the eyes that may have post-DMEK graft detachment requiring rebubbling. Larger-scale clinical trials are warranted to validate the model.

**Translational Relevance:**

MIL-AI models represent an opportunity for implementation in routine DMEK suitability screening.

## Introduction

Descemet membrane endothelial keratoplasty (DMEK) has gained vast success after its introduction by Melles et al.^1^ in 2006. However, postoperative graft detachment still remains one of the most important and common challenges in this surgery.^2^^,^^3^ Present efforts to predict graft detachments combine preoperative clinical factors and anterior segment imaging, especially anterior segment optical coherence tomography (AS-OCT), but they lack accuracy.^4^^–^^12^

Artificial intelligence (AI) in the form of deep learning is increasingly being used in the field of ophthalmology to improve diagnostic accuracy and predict pathology through pattern recognition with image analysis. Elsawy et al.^13^ developed a multipathology deep learning algorithm that identified dry eye disease, Fuchs endothelial dystrophy (FED), and keratoconus (KC) from AS-OCT images with very high performance (area under the curve [AUC] of the receiver operating characteristic [ROC] curve >0.99, F1 score >0.90). Chen et al.^14^ developed a deep learning algorithm capable of detecting various stages of KC from AS-OCT–generated maps (accuracy, 0.85–0.9). Dhommati et al.^15^ have developed an automated model to identify graft detachments after Descemet stripping automated endothelial keratoplasty (DSAEK), achieving an accuracy comparable to an expert's opinion and Dice coefficient of 81.3%. In these publications, AI models achieved similar if not better accuracy than expert identification, currently the gold standard.^13^^–^^15^

Multiple-instance learning (MIL)–based methods have been widely adopted in the task of multi-instances classification.^16^^–^^18^ Specifically, a patient level of label and “bags” or “collections” of instances (i.e., images) of the patient are available. In this type of learning, the labels are assigned to the bags, not to the instances within the bags. Each bag contains one or more instances, and at least one instance in each positive bag has the desired label. On the other hand, negative bags do not contain any instance with the desired label. Overall, MIL is useful when the label assignment is only known at the bag level, and the instances within each bag may have varying degrees of relevance to the label assignment. Such a scenario aligns with this task, and we adopt the MIL as our learning pipeline.

AI has already been used to investigate DMEK graft detachments using postoperative AS-OCT scans.^19^ However, the risk of postoperative DMEK detachment could be mitigated with OCT preoperative screening adjuvated by AI.^2^^,^^20^ To date, there is no published work on AI to investigate DMEK graft detachments using preoperative AS-OCT scans. Therefore, this study aimed to design a novel MIL-AI model to distinguish between eyes that may or may not incur a graft detachment based on pre-DMEK surgery AS-OCT images.

## Methods

### Design and Inclusion Criteria

A retrospective cohort study was performed at St. Paul's Eye Unit, Liverpool University Hospitals NHS Foundation Trust. Ethical approval was gained from the institutional review board (ID: 11392). Patient data for the study were anonymized. Patient databases were primarily screened on the local secure system to identify eyes suitable for the study. We included patients >18 years old who underwent DMEK surgery at St. Paul's Eye Unit, Liverpool University Hospitals NHS Foundation Trust in the past 4 years for FED and/or bullous keratopathy (BK). We included only patients who had performed preoperative AS-OCT imaging and whose image quality was deemed suitable for analysis. Patients with unsatisfactory imaging due to blurry images, artifacts, and incomplete visualization of the corneal endothelium were excluded. Subjects whose imaging satisfied the suitability criteria were investigated for demographics and primary/secondary outcomes.

### Imaging Protocol

Images were obtained from the AS-OCT Casia SS-1000 (Tomey, Nagoya, Japan). The proprietary high-resolution anterior segment scan, which is a radial scan covering the entire corneal surface, was employed. In particular, this scan is composed of 128 radial B-scans per eye, each with 512 A-scans (16-mm scan length).

### Data Preparation

To prepare the data for the convolutional neuronal network, each image was cropped to 1680 × 960 pixels using the auto-crop function on Microsoft Office Picture Manager 2010 (version 14.0.7010.1000; Microsoft, Redmond, WA, USA). The Office Picture Manager can auto-crop the image based on the specified pixel index (locations) and dimensions. For example, for every original image, we crop them based on the pixel index range from 32 to 996 and from 2 to 1682 along the width and height. Thus, every image follows the same index range, and we can generate the same region of interest for the cropped images. Such a process can also be easily done by programming. This allowed supplementary material captured beyond the image to be removed.^21^ Images could not be cropped any more than they were without risking losing some anterior segment capture in several images. This was due to variability in the sizes of different patients’ anterior segments.

Images were labeled into files according to if the patient had experienced/not experienced a graft detachment requiring rebubbling post-DMEK surgery. Graft detachment requiring rebubbling was defined as a detachment involving one-third of the axial extension and/or involving the central 5 mm of the cornea (pupillary area). This information was provided by postoperative OCT scans. No randomization was done as this would make no difference to the network's interpretation of the images.

The network needed data to be divided for training, validation, and testing. Images of 50 of 74 eyes were used in training and validation of the proposed model, with 20 of 50 labeled as “no detachment” and 30 of 50 labeled as “detachment.” Training network performance parameters were validated every five epochs of training. Images of the remaining 24 of 74 eyes were used for testing, with 11 of 24 labeled as “no detachment” and 13 of 24 labeled as “detachment.”

### Convolutional Neuronal Network in the MIL

**Figure 1.**
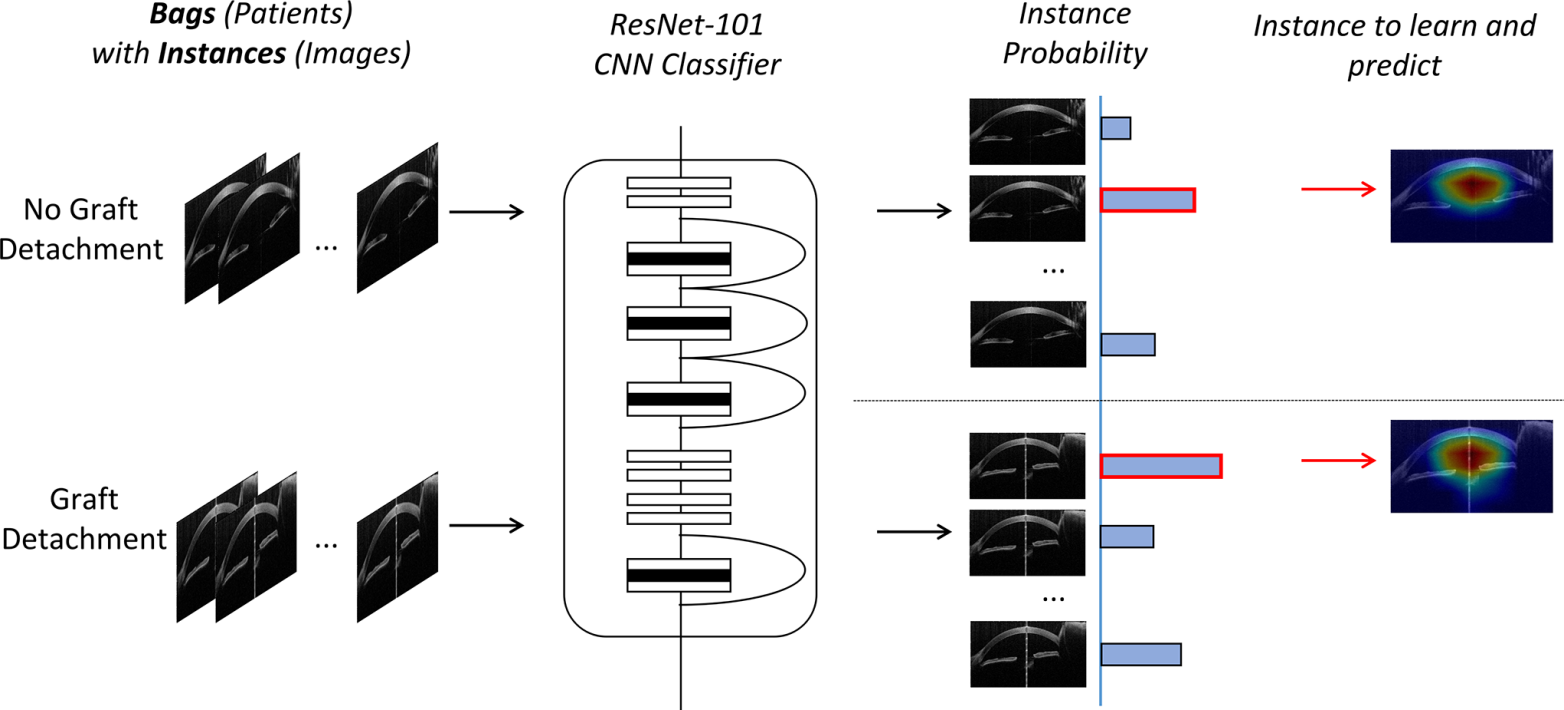
Overview of the pipeline of the MIL-AI model. Individual B-scan images of an eye are known as instances, and the whole set of B-scan images of an eye is known as a bag. Bags contain multiple instances, which are labeled as having a graft detachment or not. A graft detachment label will qualify if there was at least one instance with graft detachment in the bag. In training, bag labels are assigned to the instance labels. With training, the model recognizes patterns among instances within bags. The ResNet 101 is adopted as the backbone. The maximum-instance probability will be used for back propagation during training and be used for model prediction during inference.

Our AI model utilized MIL, and Figure 1 illustrates the proposed MIL-AI model. As shown in Figure 1, individual B-scan images of an eye are known as instances, and the whole set of B-scan images of an eye is known as a bag. Bags contain multiple instances that are labeled as having a graft detachment or not. A graft detachment label will qualify if there was at least one instance with graft detachment in the bag. In training, bag labels are assigned to the instance labels. With training, the model recognizes patterns among instances within bags. After training, the model should then be able to correctly label unseen bags.^22^ ResNet was used as the backbone classifier due to its superior performance in many classification tasks.^23^ In brief, ResNet is a convolutional neuronal network (CNN) that used residual connections for deeper neural networks training. Such a design makes it easier to learn and optimize deeper architecture of the CNN, and it addresses the issue of gradient exploration or vanishing at the same time.

In this work, different ResNet variants (ResNet-18, -34, -50, -101) have been tried to find the best model for the classification. The model selected the single image with the highest probability of correct prediction, and this was used to represent the eye. The model learned the best way to represent each eye and learned the best way to predict graft detachment.

Training involved the image being allocated a random probability of graft/no graft detachment, passing through the network, and reaching the predetermined graft/no graft detachment outcome.

### Implementation Details

A normalization process was applied to all the images so as to normalize each image with mean values of 0.5 and standard deviations to 0.5. This normalized the data pixel distribution and therefore quickened the learning time. No gamma correction was used in the normalization process. Input images were randomly transformed by on-the-fly data augmentation during training to increase sample size and reduce risks of overfitting. Transformations were up to 30° rotation, horizontal and vertical flip.

All the experiments were conducted on a local workstation with Intel Xeon W-1024 CPU and Nvidia Geforce RTX 2080Ti GPU. All the scripts, including pre- and postprocessing, were developed using PyTorch (1.13.1). A pretrained model was used due to the small size of the available data. With a small number of <10,000 images, an untrained model would often suffer from overfitting. A pretrained backbone network on ImageNet was used to prevent overfitting due to the limited data size included in this work.^24^^,^^25^ Fifty epochs were empirically chosen for the model training.

The initial weights were random probabilities between 0 and 1. In the training pipeline of the adopted instance-based multiple-instance learning, we chose one most discriminative instance per bag to represent the whole bag, which means one image per patient was automatically chosen for training. The loss function was cross-entropy loss, which is the summation of the true probability multiplied by the log-predicted probability over all classes in the distribution. The network is trained end to end by an Adam optimizer.^26^ In order to find the optimal settings of the MIL-AI model, a detailed ablation study of backbone networks was conducted, and comparisons were made between batch sizes of 2, 4, 6, and 8 and between learning rates of 0.01, 0.001, 0.0001, and 0.00001, in addition to the number of ResNet layers. Threefold cross-validation was used for tuning the hyperparameters with train and validation data sets.

### Performance Evaluation

#### HeatMaps

**Figure 2.**
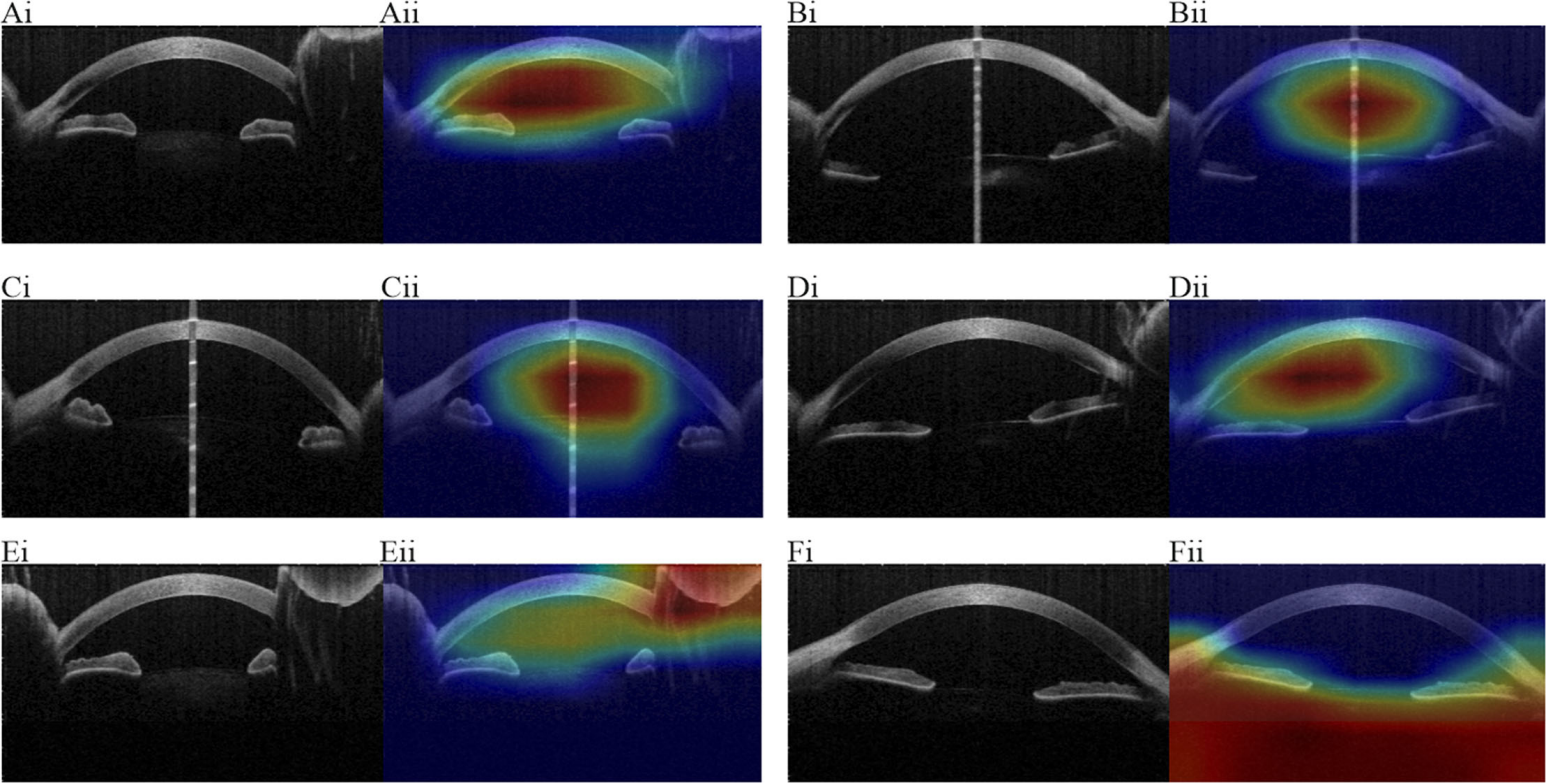
Panels show comparisons of randomly chosen pairs of original input OCT images (i) against the respective heat maps (ii). A to D are the AI looking at the correct parts of the image: (A) successful graft detachment prediction; (B) successful no graft detachment prediction; (C) unsuccessful graft detachment prediction; (D) unsuccessful no graft detachment prediction. E to F are the AI looking at the debatable parts of the image: (E) successful graft detachment prediction; (F) unsuccessful no graft detachment prediction.

Grad-CAM is an attribution method used to express the gradients at the final convolutional layer as a rough visualization, known as a heat/activation/attribution map.^27^ Figure 2 shows the pixels of the original image that affected decisions the most and visualizes these in red. The Grad-CAM in Figure 2 also uses the most discriminative gradients against the prediction and visualizes these in blue. Grad-CAM was used in this context, producing heatmaps for every test image.^28^^–^^30^

#### Performance Outcomes and Metrics

The primary outcome measure was the CNN's discrimination accuracy at predicting graft detachment/no graft detachment post-DMEK surgery, based on preoperative anterior segment OCT images. This was gauged primarily by the F1 score. The F1 score is the harmonic mean of recall (sensitivity) and precision (positive predictive value) of the model.^29^ Recall is the ratio of the number of correctly classified positives (e.g., detachment post-DMEK surgery) and the total number of positives, while precision is the ratio of correctly classified positives and the total number of predicted positives. F1 was used because the classes were imbalanced (more graft detachments than no graft detachments).

A senior cornea specialist (VR) undertook a yes/no questionnaire for graft detachment using the OCT scans and limited clinical information. Clinical factors used by the ophthalmologist to make their graft/no graft detachment predictions included age, gender, eye laterality, pre-DMEK AS-OCT timing, anterior chamber area, volume, circumference and thickness of the cornea before surgery, phacoemulsification date, graft preparation (eye bank versus surgeon stripped), folding and size, donor age and gender, endothelial cell density, and preoperative best-corrected visual acuity. AI and ophthalmologist performances were compared by prediction sensitivity, specificity, AUC, and the McNemar statistical test.

## Results

### Sample Inclusion and Exclusion

Out of 178 eyes identified during the primary screening for this study, 104 eyes were excluded while 74 eyes (69 patients) who satisfied the inclusion criteria were investigated for demographics and the primary/secondary outcomes. In total, 128 OCT images per eye should have been collected. However, due to missing scans, 127 images were collected for 6 of 74 eyes. A total of 9466 images of 74 eyes were included in this study. Forty-three of 74 eyes were labeled as “detachment.”

### Demographic Data Collection

Mean age of the patients was 68.5 ± 11.9 years. Most subjects were females (62.2%) with only 32.4% males and 5.4% with unreported gender. Of the eyes, 40.5% were right eyes. Timing of the OCT scans, relative to the operation date, spanned across a wide time range (8.6 ± 10.9 months). FED was the most common indication for surgery (FED, 87.8%; BK, 12.2%).

### Performance Evaluation

#### Primary Outcome and Model Metrics

The results of our ablation study are presented in the Table. It can be seen that ResNet-101 with a batch size of 6 and learning rate of 0.0001 yielded the best performance; as such, the classification performance of this model was analyzed hereafter unless otherwise stated. The performance results on the testing set were as follows (95% confidence interval): F1 score, 0.77 (0.57–0.91); sensitivity, 0.92 (0.73–1.00); specificity, 0.45 (0.15–0.75); precision, 0.67 (0.44–0.88); and AUC, 0.63 (0.52–0.86).

**Table.** Performance Metrics of MIL Models in Comparison

	Number of Layers									
Characteristic	18	34	50	101						
Batch size	6	6	6	2	4	6	6	**6**	6	8
Learning rate	10^−4^	10^−4^	10^−4^	10^−4^	10^−4^	10^−2^	10^−3^	**10^−^^4^**	10^−5^	10^−4^
F1 score	0.58	0.62	0.54	0.47	0.67	0.38	0.46	**0.77**	0.38	0.54
Sensitivity	0.63	0.63	0.63	0.58	0.67	0.54	0.50	**0.92**	0.54	0.63
Precision	0.67	0.62	0.78	0.76	0.67	0.29	0.54	**0.67**	0.29	0.78

Batch size, learning rate and performance metrics of the final model are highlighted in bold.

#### Heat/Activation Maps

Figure 2 shows the heatmaps for the ResNet 101, batch size 6, learning rate 10^−4^ MIL model. It shows randomly chosen pairs of original input OCT images compared against prediction heatmaps. Heatmaps are shown for successful/unsuccessful predictions for graft/no graft detachments. Most heatmaps focused on the anterior chamber and the cornea, which are expected from ophthalmologist’s intuitions. Some heatmaps showed the MIL-AI focused on the part outside of the cornea or below the cornea (Figs. 2Ei and ii, 2Fi and ii). These may be debatable areas to make graft detachment predictions.^31^

#### Ophthalmologist Prediction Metrics

**Figure 3.**
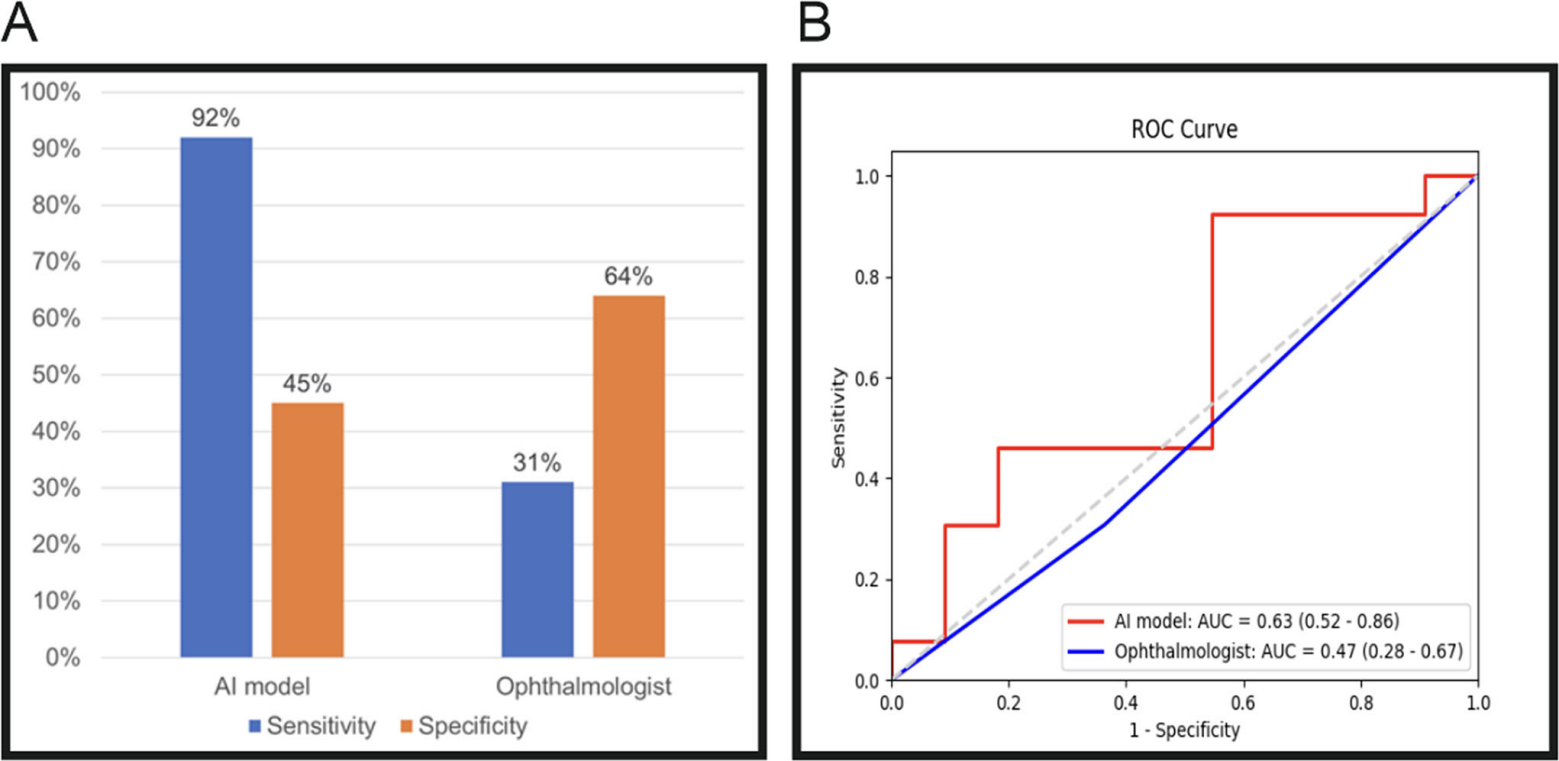
Overall ophthalmologist performance metrics compared to the MIL-AI: (A) sensitivity and specificity for discrete comparison; (B) ROC curves for continuous comparison.

The ophthalmologist predicted graft detachments to an overall worse sensitivity, but better specificity, compared to the MIL-AI. Figure 3A shows the ophthalmologist performance compared to the AI performance in terms of sensitivity and specificity. Figure 3B shows the ophthalmologist's ROC curve and corresponding AUC compared to that of the AI model during testing. The results of the McNemar test showed a statistically significant superiority of the MIL-AI model against the ophthalmologist's predictions (*P* < 0.05).

## Discussion

In this article, we elaborated and validated a deep learning convolutional neural network, MIL-AI, capable of predicting the probability of post-DMEK graft detachment from preoperative AS-OCT scans. The model performed with very high sensitivity and relatively low specificity, with overall better evaluation performance metrics than those of a senior ophthalmologist provided with clinical and surgical data and preoperative imaging.

DMEK is a relatively new, complex, but increasingly popular surgical approach to endothelial failure.^2^ In order to identify ideal candidates for DMEK, an algorithm capable of accurate prediction of immediate postoperative complications such as detachment rate would make for a useful screening tool.

Overall, a growing body of evidence supports the use of AI-based pre- and postoperative screening as a tool that could help with predicting and diagnosing pathologic consequences of surgery.^14^^,^^19^^,^^30^^,^^32^^,^^33^ Deep learning models have been employed to identify postoperative DMEK graft detachments,^19^ to predict future need and suitability for keratoplasty,^30^ to quantify DMEK graft detachment segmentation,^32^ to identify the best predictors for graft detachment after endothelial keratoplasty,^33^ and to predict the need for rebubbling after DMEK surgery.^34^ Indeed, clinical trials are currently starting to incorporate AI into corneal pathology identification. The European Vision Institute stated that graft detachment could be objectively quantified with AI and that such standardized data should be used to report outcomes for endothelial keratoplasties.^20^^,^^35^

This study represents a novel approach to DMEK detachment analysis as it focused solely on preoperative AS-OCT imaging. A preoperative algorithm such as the one we developed could provide the ophthalmologist with a valuable screening tool, because with a high-sensitivity MIL-AI, subjects at high risk for DMEK graft detachment could be confidently identified and monitored. Hayashi and colleagues^34^ have employed machine learning on postoperative AS-OCT images to predict whether a DMEK would need/not need to be rebubbled due to a graft detachment. Although the present study achieved lower accuracy, our algorithm still performed better than an ophthalmologist provided with clinical and imaging data, proving that it could be of help in correctly identifying high-risk eyes. In addition, the proposed AI model has only used images for the prediction, whereas the ophthalmologist had full access to the data, which may not be a fair comparison for the AI model. On the other hand, this suggests that the MIL-AI model may be further improved if it can take clinical and demographic data into account in the future.

Larger-scale trials should be undertaken using this MIL-AI model for external validation before it is adopted into clinical practice. Successful trials will warrant planning to assess cost-effectiveness for an economically viable implementation of this technology into everyday clinical practice. Limitations of the study are mostly inherent to the small sample size. A drawback of automated models is the risk of error in smaller samples. This is sometimes mitigated by manual image labeling. This study was restricted in this respect, with a limited sample size mitigated by a supervised learning model. In this study, we chose to utilize a multiple-instance learning model. This kind of supervised learning is extensively used in the medical imaging field, as it does not require extensive manual labeling of single scans (instances) but rather of the entirety of the radial AS-OCT volume (bag).^22^

The sensitivity of our model was higher than the specificity. This means that the MIL-AI model tended to generate more false-positive results rather than false-negatives results. However, higher sensitivity is warranted in screening tools rather than specificity, as it ensures that as little cases likely to experience the outcome as possible are missed. In this case, since the model could be employed to screen patients scheduled for DMEK, identifying subjects at risk for postoperative graft detachment with sufficient sensitivity is a priority.

In conclusion, we observed that a self-designed, supervised MIL-AI could successfully use preoperative AS-OCT scans to predict patients who would have DMEK graft detachments with reasonable accuracy. Although larger-scale clinical trials to externally validate this model are warranted, the MIL-AI model represents an opportunity to improve preoperative DMEK suitability screening.
